# Tackle Height and Tackle Success—An Analysis of 52,204 Tackle Events

**DOI:** 10.1002/ejsc.70003

**Published:** 2025-07-12

**Authors:** S. Hendricks, K. Till, S. Scantlebury, N. Dalton‐Barron, S. den Hollander, N. Gill, S. Kemp, A. Kilding, M. Lambert, P. Mackreth, J. O’Reilly, C. Owen, K. Spencer, K. Stokes, J. Tee, R. Tucker, L. Vaz, D. Weaving, S. W. West, K. Dane, F. McKnight, B. Jones

**Affiliations:** ^1^ Division of Physiological Sciences and Health Through Physical Activity, Lifestyle and Sport Research Centre Department of Human Biology Faculty of Health Sciences University of Cape Town Cape Town South Africa; ^2^ Carnegie Applied Rugby Research (CARR) Centre, Carnegie School of Sport Leeds Beckett University Leeds UK; ^3^ England Performance Unit Rugby Football League Manchester UK; ^4^ Division of Health, Engineering, Computing and Science Te Huataki Waiora School of Health University of Waikato Tauranga New Zealand; ^5^ Rugby Football Union London UK; ^6^ London School of Hygiene and Tropical Medicine London UK; ^7^ Sports Performance Research Institute New Zealand Auckland University of Technology Auckland New Zealand; ^8^ Department of Sports Science and Physical Education The Chinese University of Hong Kong Shatin Hong Kong; ^9^ Centre for Health, Injury and Illness Prevention in Sport University of Bath Bath UK; ^10^ UK Collaborating Centre on Injury and Illness Prevention in Sport University of Bath Bath UK; ^11^ World Rugby Dublin Ireland; ^12^ Research Center in Sports Sciences, Health Sciences and Human Development (CIDESD) University of Trás‐os‐Montes and Alto Douro Vila Real Portugal; ^13^ Sport Injury Prevention Research Centre Faculty of Kinesiology University of Calgary Calgary Canada; ^14^ Premiership Rugby London UK; ^15^ School of Behavioural and Health Sciences, Faculty of Health Sciences Australian Catholic University Brisbane Australia

**Keywords:** game analysis, injury and prevention, performance, skill, team sport

## Abstract

To compare the probability of tackle success (the tackler preventing the ball‐carrier and ball from progressing towards the tackler try‐line) when contacting the ball‐carrier at different heights (shoulder, mid‐torso and legs) for different types of tackles (active, passive, smother and arm) while accounting for other tackler situational factors within seven playing levels. Video footage of 271 male rugby union matches were analysed across seven playing groups (Under [U] 12, *n* = 25 matches; U14, *n* = 35; U16, *n* = 39; U18 Amateur *n* = 39; U18 Elite *n* = 38; Senior Amateur, *n* = 40 and Senior Elite, *n* = 50) across England, New Zealand, South Africa, Portugal and USA (a total of 51,106 tackles). A multi‐level logistic regression model with tackle success as the outcome variable and first point of contact and type of tackle as the explanatory variables were computed. Included in the model as cofounders were the situational variables tackle direction, tackle sequence, number of players in the tackle and attacker intention. Post‐estimation marginal effects were used to calculate the probabilities (expressed as a percentage %) of tackle success for each interaction between tackle type (active shoulder, smother, passive shoulder and arm) and the first point of contact (shoulder, mid‐torso and legs). The probability of tackle success in relation to where the ball‐carrier is contacted varied by tackle type and within each age group. The probabilities (Pr) for contacting the shoulder versus mid‐torso at the senior levels (elite and amateur) did not differ in relation to tackle success (for instance, for active shoulder tackles within senior elite; shoulder Pr 86% 95% CI 82–89 and mid‐torso Pr 82% 95% CI 77–86), whereas at the junior levels, contacting the shoulder had a higher probability than other points of contact. Active shoulder tackles had the highest probability of tackle success across the different playing levels across the different contact heights, whereas arm tackles had the lowest probability (for instance, for mid‐torso tackles within senior elite, active Pr 82% 95% CI 77–86 vs. arm Pr 69% 95% CI 64–75). Coaches and practitioners can use this information to improve tackle training design and planning within the different age groups and facilitate player development.

## Summary

- From a performance perspective, contacting the shoulder versus mid‐torso at the senior levels (elite and amateur) made no difference in tackle outcomes.
- At the junior levels, there was a small advantage for contacting the shoulder, which likely relates to weaker tackling technique and attempts to prevent offloads.
- Active shoulder tackles had the highest probability of tackle success across all playing levels, irrespective of contact height, whereas arm tackles had the lowest probability.
- The probability of tackle success in relation to where the ball‐carrier is contacted varied by tackle type and within each age group, highlighting that there are different approaches to ‘win’ the tackle contest.

## 1 Introduction

Rugby union (henceforth called rugby) is one of the world's most popular team sports, with approximately 7.6 million players in 128 countries (World Rugby 2021) playing professionally and recreationally. Rugby is played from junior to senior levels and consists of both male and female competitions. At the junior levels, the starting age group for participation in rugby depends on the country but generally may be as young as 7 years of age. Boys and girls may play together up to a certain age group within different countries. The inclusion of the tackle in the introductory format of rugby also differs between countries. In a study combining male data from three rugby playing nations, a tackle occurred 155 times on average during an under 12 match, 204 times at the under 18 level and 221 times on average during a senior elite level match (Till et al. 2023). The frequency of the tackle at the different age groups highlights that participation and performance in rugby is reliant on player's ability to safely and successfully contest the tackle.

Whether as the ball‐carrier or the tackler, performance in rugby is dependent on a players' ratio of successful and unsuccessful tackles (Paul et al. 2022; Burger et al. 2020; Till et al. 2023; Watson et al. 2017). In addition to the performance demands, the frequent and dynamic physical–technical and psychological nature of the tackle exposes players to risk of injury. Tackle injuries are responsible for the highest proportion of all injuries, highest injury incidence and highest injury burden (Burger et al. 2020; S. Williams et al. 2022; Yeomans et al. 2018; West et al. 2021; Brooks et al. 2005; West et al. 2023). The tackle contest is also the leading cause of head injuries including concussion (West et al. 2023; Mc Fie et al. 2016; Roberts et al. 2017; van Tonder et al. 2023; Gardner et al. 2014). Reducing head injury and concussion risk, at all levels, is a top priority for rugby stakeholders and governing bodies (Hendricks et al. 2023). Based on video analyses of head injuries and concussion, players tackling technique has been identified a major injury risk factor (Davidow et al. 2018; Tierney et al. 2018; Tierney and Simms 2018; Hendricks et al. 2016; Hendricks, O’connor, et al. 2015; Tucker et al. 2017). Specifically, the height where the tackler makes the initial contact on the ball‐carrier has shown to significantly change players' concussion injury risk (Davidow et al. 2018; Tierney and Simms 2018; Tucker et al. 2017). It is also well established that tackling technique is dependent on other situational factors such as the type of tackle, tackle direction and attacking intention (the running pattern of ball‐carrier before the tackle) (Hendricks et al. 2016; Tucker et al. 2017; Burger et al. 2016; Fuller et al. 2010; Quarrie and Hopkins 2008). As such, these situational factors should be accounted for when analysing tackle height.

A low body‐position and aiming for the ball‐carrier's centre of gravity have always formed part of rugby injury prevention education for coaching the tackle (Hendricks and Lambert 2010). Recently, in an effort to change player's on‐field tackler behaviours to be in line with these recommendations and contact the ball‐carrier lower, World Rugby has implemented stricter rulings around high‐tackles and trialled law changes to lower the height of a legal tackle to below the bottom of the sternum (van Tonder et al. 2023; Raftery et al. 2021; Stokes et al. 2021). In the wake of these law change trials, new tackle laws where the height of a legal tackle is below the bottom of the sternum have been implemented at the community and junior levels across all major rugby playing nations (Hendricks et al. 2024). Technique interventions and tackle programmes that focus on improving player's tackling ability have also been proposed (Hendricks, Till, et al. 2018; Davidow et al. 2023; Edwards et al. 2021; Hendricks et al. 2022). It is well‐understood that an integrated collective approach that includes both active (e.g., training interventions and coach education) and passive measures (e.g., law changes) is required to prevent head injury and concussion (Burger et al. 2020; Hendricks et al. 2023; Eliason et al. 2023). However, the success of these head injury prevention strategies is, in part, reliant upon its adoption by coaches and players, which is likely influenced by its association with performance (Finch 2012; Hendricks, den Hollander, et al. 2015; Hendricks et al. 2012). As such, understanding the performance changes when contacting the ball‐carrier at different tackle heights, while accounting for other situational factors, may help with the overall implementation of a tackle‐height law change (Hendricks et al. 2024). In other words, governing bodies can address any performance change concerns coaches may have related to the tackle‐height law modifications.

The majority of studies associating tackle characteristics with performance have been conducted on senior elite players, with no studies to date investigating tackle characteristics associated with performance at the different levels of play, specifically junior levels (Hollander et al. 2021). As mentioned, tackle performance, or tackle success, within video analysis studies is typically defined based on the progress of the ball‐carrier and ball towards the opposition try‐line (Hendricks et al. 2020; den Hollander et al. 2018; Hendricks, van Niekerk, et al. 2018; Hendricks et al. 2014). Considering the tackle height law is in the early stages of implementation and evaluation in some countries, and going to be implemented across the globe, understanding the performance changes when contacting the ball‐carrier at different tackle heights at the different levels of play will significantly contribute to the collective project of reducing head injury and concussion in rugby.

At all levels of play, the three main tackle injury prevention strategies in rugby are law changes and referee application, coach education and implementing tackle‐specific training programmes. Within each of these tackle injury prevention strategies, a key technical recommendation is where to contact the ball‐carrier (tackle height). To promote this technical recommendation and enhance its perceived attributes within the injury prevention strategies (Hendricks et al. 2024), understanding the relationship between tackle height and tackle success while accounting for other tackle situational factors at the different levels of play is warranted. Therefore, the purpose of this study is to compare the probability of tackler success when contacting the ball‐carrier at different heights (shoulder, mid‐torso and legs) for different types of tackles (active, smother, passive and arm) while accounting for other tackler situational factors, within seven male playing levels (Under 12, Under 14, Under 16, Under 18 Amateur, U18 Elite, Senior Amateur and Senior Elite).

## 2 Methods

This study formed part of a larger project to understand collision and noncollision match characteristics at different levels of play (Till et al. 2023). Video footage of 271 male rugby matches were collected and analysed across five age categories (U12, U14, U16, U18 and Senior) and two playing standards (Amateur and Elite) within England, New Zealand, South Africa, Portugal and USA. Amateur playing standards included education and community (i.e., competitive match‐played between two teams of players representing their school, university or an amateur team) rugby matches. Elite playing standards included international, professional (i.e., competitive match‐played between two international teams or teams at the highest standard where players are paid to play) and Under 18 Elite (highest standard but not paid to play) rugby matches. This resulted in seven independent playing groups (i.e., U12, *n* = 25 matches; U14, *n* = 35; U16, *n* = 39; U18 Amateur *n* = 39; U18 Elite *n* = 38; Senior Amateur, *n* = 40 and Senior Elite, *n* = 50). It should be acknowledged that player numbers (e.g., U12 = 12‐a‐side vs. U14 and above = 15‐a‐side), pitch size (e.g., U12 = 70 × 50 m vs. U14 and above = full size), playing duration (e.g., U12 = 40 min vs. Senior = 80 min) and playing rules (e.g., U12 = uncontested scrums, U14 = uncontested lineouts) were not the same for each playing group. However, all analyses were conducted within each playing group; therefore, these differences had little effect on the analyses. The number of tackles within each playing group were U12 = 4,047, U14 = 5,348, U16 = 6,846, U18 Amateur = 7931, U18 Elite = 7677, Senior Amateur = 8338 and Senior Elite = 10,919.

### 2.1 Protocol

All analyses were performed at the match level with no coding of individual players. All matches were competitive and played between 2017 and 2019, adopting the laws of World Rugby. Matches were screened for suitability to meet the criteria (i.e., complete match, appropriate age category and playing standard within each country). All video recordings of matches were obtained from a principal investigator from each country, and it was their responsibility to source the video footage of matches from existing recorded matches or by filming matches prospectively. All match footage were screened for completeness and quality by the lead analyst. The quality of the video footage was considered suitable when match events were clearly visible and interpretable. Match footage was predominantly filmed from one camera angle at an elevated side position at the halfway line. This allowed the camera to follow the ball during play and zoom in on specific match events. Match footage were excluded if the angle of the footage was too wide, too high or unclear to accurately code. Insufficient footage quality contributed to a lower sample size at the U12 and U14 levels as factors such as camera position restricted the clarity of match events. Ethics approval was obtained for the filming and analysis of matches in line with Helsinki International ethics. Consent for the use of the videos and analysis was provided by national governing bodies and a representative from each team.

Match video footage was analysed using Sports Code Elite Version 14 (Sportstec), using an Apple iMac or Macbook (Apple Inc., Cupertino, California USA). The analysis software allowed control over the speed at which each movement could be viewed and the recording and saving of each coded instance into a database. During the analyses, the analyst could pause, rewind and watch the footage in slow motion. The highest frame frequency the analyst could slow down the motion of the footage was to 25 frames per second. Match characteristics were coded by nine video analysts based in two laboratories (*n* = 4 Leeds, England and *n* = 5 Cape Town, South Africa). To enhance consistency between analysts, the lead analyst from the two video analysis laboratories collaboratively reviewed a full match examining each match characteristic and their associated definitions. During the training process, each match characteristic was replayed at 25 frames per second to facilitate a clear distinction between coding criteria. The initial training process lasted approximately six hours with 15 min breaks incorporated every hour. The lead analysts repeated this process with the remaining seven analysts from their respective video analysis laboratories until each analyst understood the coding process for each variable. If an analyst was unclear on the coding process for a match event, an online meeting was arranged between the video analysis laboratories until a resolution was established. Once each analyst indicated they understood the variables and definitions, they were tested for intra‐ and inter‐rater reliability and these results have been reported elsewhere (Till et al. 2023). For the tackle, the intra‐reliability Kappa mean was 0.80 (95% CI 0.73–0.88), which is considered ‘substantial agreement’, and the inter‐reliability Kappa mean was 0.70 (95% CI 0.68–0.73), which is also considered ‘substantial agreement’ (Till et al. 2023).

Match characteristics were coded using the definitions established by the Rugby Union Video Analysis Consensus group (Hendricks et al. 2020). For this study specifically, tackle success, tackle type, tackle direction, tackle point of contact, tackle sequence, number of players and attacking intention were used (Table 1). In line with previous work and the rugby video analysis consensus (Hendricks et al. 2020; den Hollander et al. 2018; Hendricks, van Niekerk, et al. 2018; Hendricks et al. 2014), a successful tackle for this study was observed when ‘the tackler prevents the ball‐carrier and ball from progressing towards his try‐line that is prevents an offload of the ball, tackle‐break or try, and the tackler does not concede a penalty’. An unsuccessful tackle was observed when ‘the ball‐carrier and ball progressed towards the tackler try‐line that is the ball‐carrier was able to offload the ball, break the attempted tackle, or scores a try, or the tackler concedes a penalty’.

**TABLE 1:** Tackle variables and definitions.

Variable	Operational definition
Tackle event	An event where one or more tacklers (player or players making the tackle) attempted to stop or impede the ball‐carrier (player carrying the ball) whether or not the ball‐carrier was brought to ground.
Tackle type	*Arm tackle* Tackler impedes ball‐carrier with upper limbs. *Shoulder active tackle* First contact is with the tackler's shoulder, and the tackler drives or attempts to drive the ball‐carrier backwards. *Shoulder passive tackle* First contact is with the tackler's shoulder, and the tackler does not drive or attempts to drive the ball‐carrier backwards. *Smother tackle* Tackler uses chest and wraps both arms around ball‐carrier.
Tackle direction	*Behind* Tackler makes contact with the ball‐carrier's from behind. *Front* Tackler makes contact with the front of the ball‐carrier. *Oblique* Tackler makes contact with ball‐carrier at an angle *Side* Tackler makes contact with the ball‐carrier's side.
Point of (first) contact	*Head and neck* Above the shoulder (shirt/neck) with any connection with the head/neck during the course of the tackle. *Leg* Area below the hips (shorts line) *Mid‐torso* Above the ball‐carrier's hip level (shorts line) to the level of the ball‐carrier's arm pit. *Shoulder* From the ball‐carrier's arm pit level to the shoulder.
Sequencing	*Attacking sequential* One attacker contacts one defender, followed by a second attacker joining the contact situation. *One‐on‐one* One defender contacts one attacker. *Sequential* One defender contacts one attacker, followed by a second defender joining the contact situation. (can be coded as a separate tackle) *Simultaneous* Two defenders contact one attacker at the same time (coded as separate tackles)
Attacking intention	*Arcing run* Ball‐carrier performs an arcing run. *Diagonal run* Ball‐carrier runs at an angle, instead of straight at the tackler. *Lateral run* Ball‐carrier performed a run from touchline to touchline. *Side step* Ball‐carrier performed an evasive step initiated by either leg before contact. *Straight* Ball‐carrier ran straight at the defence.
Tackle outcomes	S*uccessful* When the tackler prevents the ball‐carrier and ball from progressing towards his try‐line, that is, the tackler prevents an offload of the ball, tackle‐break or try and the tackler does not concede a penalty *Unsuccessful* When the ball‐carrier and ball progressed towards the tackler try‐line, that is, the ball‐carrier was able to offload the ball, break the attempted tackle, or scores a try or the tackler concedes a penalty

### 2.2 Statistical Analyses

A multi‐level logistic regression model with tackle success as the outcome variable and first point of contact and type of tackle as the explanatory variables were computed. Included in the model as covariates were the situational variables tackle direction, tackle sequence, number of players in the tackle and attacker intention. The interaction between first point of contact and type of tackle type was captured during post‐estimation. To account for clustering, ‘country’ and ‘match’ were included in the model as random effects. A model for each age group was computed separately.

Post‐estimation marginal predictions were computed to estimate the adjusted probability of tackle success for each interaction between tackle type (active shoulder, smother, passive shoulder and arm) and the first point of contact (shoulder, mid‐torso and legs; R. Williams 2012). These predictions were derived from a multilevel regression model that included tackle type, point of contact and relevant situational variables.

The *margins* command in Stata was used to estimate adjusted probabilities (Pr), expressed as percentages, for a given explanatory variable (e.g., tackle type), while holding the other explanatory variable (e.g., point of contact) fixed at a specific level (e.g., mid‐torso) (R. Williams 2012). All situational covariates were held at their observed values using the *asobserved* option, ensuring that the resulting marginal predictions reflected the actual distribution of these variables within the sample (R. Williams 2012). This approach yielded more realistic population‐averaged estimates of tackle success.

The results were retained for subsequent contrast testing using the post option, and coefficient labels were preserved (*coeflegend*) to facilitate interpretation. The *lincom* command was then used to test for statistically significant differences between specific predicted margins. An a priori alpha level of *p* < 0.05 was used to determine statistical significance. Probabilities and their 95% confidence intervals (95% CIs) were reported as percentages (rounded off to the nearest integer), as these are more intuitive and meaningful for interpretation than odds ratios (R. Williams 2012). A point estimate probability ≥ 5% was considered meaningful and highlighted in blue in the tables.

In the text, the contact point by type of tackle and the type of tackle by contact point, with the significantly highest or similar (not‐significantly different) probabilities, summarised for each age group. The probability margins (including 95% CI) and difference in probabilities (including 95% CI) for each contact point by type of tackle and the type of tackle by contact point are also reported for each age group. The magnitude of the difference remains the same regardless of the order of comparison (e.g., shoulder vs. leg is equivalent to leg vs. shoulder in absolute value); therefore, only three unique pairwise comparisons are reported for point of contact and six unique pairwise comparisons are reported for type of tackle. The sign of the difference (positive or negative) reflects the direction of subtraction. All statistics were computed using STATA 18 (StataCorp, College Station, TX, USA).

## 3 Results

Table 2 shows the Pr for tackle success for each tackle type and tackle height. Comparing the three tackle heights within each age group, the contact point with the highest probability of success at the Senior Elite level was the *shoulder area*, followed by the *mid‐torso* (Table 3). At the Senior Amateur and U16 levels, the contact points with the highest probability of success were the *shoulder area*, followed by the *legs* (Table 3). For both U18 levels (Elite and Amateur), the contact point with the highest probability of success was the *shoulder area*. At the U14 and U12 levels, it was the *shoulder area* and the *legs* (Table 3).

**TABLE 2:** Probability margins (Pr) of tackle success for each tackle type and tackle height.

	Arm			Active			Passive			Smother		
	Pr	−95% CI	+95% CI	Pr	−95% CI	+95% CI	Pr	−95% CI	+95% CI	Pr	−95% CI	+95% CI
Senior Elite												
Shoulder	75	70	80	86	82	89	84	80	88	87	84	91
Mid‐torso	69	64	75	82	77	86	80	75	84	84	80	88
Leg	65	59	71	78	74	83	76	71	82	81	76	85
Senior Amateur												
Shoulder	77	75	79	90	88	92	84	81	86	90	88	92
Mid‐torso	71	69	74	86	83	88	86	83	88	86	83	90
Leg	74	71	77	88	85	90	81	78	84	88	85	91
U18 Elite												
Shoulder	70	66	74	85	82	88	78	75	82	86	83	89
Mid‐torso	63	59	67	80	77	83	72	68	76	81	78	84
Leg	62	57	66	79	75	82	71	67	75	80	76	84
U18 Amateur												
Shoulder	72	69	76	87	85	90	77	73	81	84	81	88
Mid‐torso	66	62	69	83	80	86	71	67	75	79	75	83
Leg	67	63	71	84	81	87	72	68	76	80	76	84
U16												
Shoulder	67	63	71	85	82	89	76	71	80	88	84	91
Mid‐torso	59	54	63	79	76	83	68	63	72	82	78	86
Leg	62	58	67	82	79	86	71	67	76	85	81	89
U14												
Shoulder	63	57	69	89	85	93	71	64	78	80	74	85
Mid‐torso	56	49	62	85	80	90	64	57	71	73	67	80
Leg	61	54	67	88	84	92	69	62	75	78	71	84
U12												
Shoulder	52	45	58	77	71	84	68	61	75	70	63	77
Mid‐torso	47	41	54	74	67	80	64	56	72	66	59	74
Leg	54	47	61	79	73	85	70	63	78	73	65	80

*Note:* Data expressed as a probability percentage (%) along with 95% confidence intervals (95% CIs).

**TABLE 3:** Percentage differences in probability (%) of tackle success and 95% confidence interval (95% CI) between tackle heights by tackle type.

	Senior Elite		Senior Amateur		U18 Elite		U18 Amateur		U16		U14		U12	
(a) By first contact point	Difference (95% CI)	*p* value	Difference (95% CI)	*p* value	Difference (95% CI)	*p* value	Difference (95% CI)	*p* value	Difference (95% CI)	*p* value	Difference (95% CI)	*p* value	Difference (95% CI)	*p* value
*Arm Tackles*														
Shoulder versus mid‐torso	**5 (3 to 8)**	**< 0.001**	**6 (4 to 8)**	**< 0.001**	**7 (5 to 10)**	**< 0.001**	**7 (4 to 9)**	**< 0.001**	**9 (6 to 11)**	**< 0.001**	**8 (5 to 11)**	**< 0.001**	**4 (1 to 7)**	**< 0.01**
Shoulder versus leg	**9 (7 to 12)**	**< 0.001**	**3 (1 to 6)**	**0.016**	**9 (6 to 12)**	**< 0.001**	**5 (3 to 8)**	**< 0.001**	**5 (2 to 8)**	**0.004**	3 (1 to 6)	**0.160**	−3 (−7 to −2)	0.223
Mid‐torso versus leg	**4 (2 to 6)**	**< 0.001**	**−3 (**−**5 to 0)**	**0.032**	1 (1 to 4)	0.318	1 (1 to 4)	0.307	**−4 (**−**7 to** −**1)**	**0.011**	**−5 (**−**9 to** −**2)**	**0.005**	**−7 (**−**11 to** −**3)**	**0.001**
*Active shoulder tackles*														
Shoulder versus mid‐torso	**4 (2 to 6)**	**< 0.001**	**4 (2 to 5)**	**< 0.001**	**5 (3 to 7)**	**< 0.001**	**5 (3 to 6)**	**< 0.001**	**6 (4 to 8)**	**< 0.001**	**4 (2 to 6)**	**< 0.001**	**4 (1 to 6)**	**0.011**
Shoulder versus leg	**7 (5 to 9)**	**< 0.001**	2 (0 to 3)	**0.016**	**6 (4 to 8)**	**< 0.001**	**4 (2 to 5)**	**< 0.001**	**4 (1 to 7)**	**0.004**	**1 (1 to 3)**	**0.163**	−2 (−5 to 1)	0.224
Mid‐torso versus leg	**3 (1 to 5)**	**0.001**	**−2 (**−**3 to 0)**	**0.033**	1 (1 to 3)	0.320	1 (1 to 3)	0.307	**−4 (**−**6 to** −**1)**	**0.012**	**−3 (**−**5 to** −**1)**	**0.007**	**−6 (**−**9 to 2)**	**0.002**
*Passive tackles*														
Shoulder versus mid‐torso	**4 (3 to 6)**	**< 0.001**	**5 (3 to 7)**	**< 0.001**	**6 (4 to 9)**	**< 0.001**	**6 (4 to 9)**	**< 0.001**	**8 (6 to 10)**	**< 0.001**	**7 (4 to 10)**	**< 0.001**	**4 (1 to 7)**	**0.01**
Shoulder versus leg	**8 (5 to 10)**	**< 0.001**	2 (1 to 5)	0.015	**8 (5 to 10)**	**< 0.001**	**5 (2 to 8)**	**< 0.001**	**4 (1 to 7)**	**0.003**	**2 (1 to 6)**	**0.158**	−2 (−6 to 1)	0.224
Mid‐torso versus leg	**3 (1 to 5)**	**< 0.001**	**−2 (**−**5 to 0)**	**0.033**	1 (1 to 4)	0.318	1 (1 to 4)	0.309	**−4 (**−**6 to** −**1)**	**0.011**	**−5 (**−**8 to** −**1)**	**0.005**	**−6 (**−**10 to** −**3)**	**0.001**
*Smother tackles*														
Shoulder versus mid‐torso	**4 (2 to 5)**	**< 0.001**	**4 (2 to 5)**	**< 0.001**	**5 (3 to 7)**	**< 0.001**	**5 (3 to 7)**	**< 0.001**	**5 (4 to 7)**	**< 0.001**	**6 (3 to 9)**	**< 0.001**	**4 (1 to 7)**	**0.01**
Shoulder versus leg	**7 (5 to 9)**	**< 0.001**	2 (0 to 3)	**0.021**	**6 (4 to 8)**	**< 0.001**	**4 (2 to 6)**	**< 0.001**	**3 (1 to 5)**	**0.007**	**2 (1 to 5)**	**0.168**	−2 (−6 to −1)	0.219
Mid‐torso versus leg	**3 (1 to 5)**	**0.001**	**−2 (**−**3 to 0)**	**0.032**	1 (1 to 3)	0.320	1 (1 to 3)	0.305	**−3 (**−**5 to** −**1)**	**0.011**	**−4 (**−**7 to** −**1)**	**0.005**	**−6 (**−**10 to 2)**	**0.001**

*Note: p* values are also shown for each comparison, with significant differences bolded. Percentage differences more than 5% highlighted in blue.

Comparing the four tackle types within each age group, *arm tackles* consistently demonstrated the lowest probability of tackle success as indicated by ≥ 5% positive percentage differences when compared with other tackle types and highlighted in blue in Table 4. These differences were statistically significant across all *arm tackle* comparisons (*p* < 0.001), with confidence intervals consistently excluding zero. Among the Senior Elite, Senior Amateur, U18 Elite, U18 Amateur and U16 groups, the highest probabilities of success were observed for *active shoulder* and *smother tackles*, with small and nonsignificant differences between them (*active* vs. *smother*: range −1% to 4% and *p* > 0.05). In contrast, in the U14 and U12 groups, *active shoulder* tackles alone demonstrated the highest probability of success.

**TABLE 4:** Percentage differences in probability (%) of tackle success and 95% confidence interval (95% CI) between tackle types by tackle height.

	Senior Elite		Senior Amateur		U18 Elite		U18 Amateur		U16		U14		U12	
(b) By type of tackle	Difference (%)	*p* value	Difference (%)	*p* value	Difference (%)	*p* value	Difference (%)	*p* value	Difference (%)	*p* value	Difference (%)	*p* value	Difference (%)	*p* value
*Shoulder*														
Active versus passive	**2 (0 to 4)**	**0.126**	**6 (3 to 8**)	**< 0.001**	**7 (4 to 9)**	**< 0.001**	**10 (7 to 13)**	**< 0.001**	**10 (6 to 13)**	**< 0.001**	**18 (13 to 23)**	**< 0.001**	**9 (3 to 15)**	**0.002**
Active versus smother	−2 (0 to −4)	0.082	0 (−3 to 2)	0.773	−1 (−3 to 2)	0.487	**3 (0 to 6)**	**0.043**	−2 (−5 to −1)	0.204	**9 (5 to 14)**	**< 0.001**	**7 (1 to 13)**	**0.019**
Active versus arm	**11 (8 to 13)**	**< 0.001**	**12 (10 to 15)**	**< 0.001**	**15 (12 to 18)**	**< 0.001**	**15 (13 to 17)**	**< 0.001**	**18 (15 to 21)**	**< 0.001**	**26 (22 to 30)**	**< 0.001**	**26 (21 to 30)**	**< 0.001**
Smother versus passive	**3 (1 to 5)**	**0.002**	**6 (3 to 9)**	**< 0.001**	**7 (5 to 10)**	**< 0.001**	**7 (3 to 11)**	**< 0.001**	**12 (8 to 16)**	**< 0.001**	**9 (3 to 14)**	**0.002**	2 (4 to 9)	0.486
Smother versus arm	**13 (10 to 15)**	**< 0.001**	**13 (10 to 16)**	**< 0.001**	**16 (13 to 18)**	**< 0.001**	**12 (8 to 15)**	**< 0.001**	**20 (17 to 24)**	**< 0.001**	**16 (12 to 21)**	**< 0.001**	**19 (14 to 23)**	**< 0.001**
Passive versus arm	**9 (7 to 12)**	**< 0.001**	**7 (4 to 9)**	**< 0.001**	**8 (6 to 11)**	**< 0.001**	**5 (2 to 7)**	**0.001**	**8 (5 to 11)**	**< 0.001**	**8 (4 to 12)**	**< 0.001**	**17 (11 to 22)**	**< 0.001**
*Mid‐torso*														
Active versus passive	**2 (0 to 4)**	**0.125**	**7 (4 to 10)**	**< 0.001**	**8 (5 to 11)**	**< 0.001**	**12 (9 to 16)**	**< 0.001**	**12 (8 to 16)**	**< 0.001**	**21 (16 to 26)**	**< 0.001**	**10 (3 to 16)**	**0.002**
Active versus smother	−2 (0 to −4)	0.079	−1 (−4 to 3)	0.773	−1 (−4 to 2)	0.486	**4 (0 to 8)**	**0.043**	−3 (−7 to −1)	0.202	**11 (6 to 17)**	**< 0.001**	**7 (1 to 14)**	**0.019**
Active versus arm	**12 (10 to 15)**	**< 0.001**	**15 (12 to 17)**	**< 0.001**	**15 (12 to 18)**	**< 0.001**	**17 (15 to 20)**	**< 0.001**	**21 (18 to 24)**	**< 0.001**	**29 (25 to 34)**	**< 0.001**	**26 (22 to 31)**	**< 0.001**
Smother versus passive	**4 (2 to 6)**	**0.001**	**8 (4 to 11)**	**< 0.001**	**9 (6 to 12)**	**< 0.001**	**8 (4 to 13)**	**< 0.001**	**14 (10 to 19)**	**< 0.001**	10 (3 to 16)	0.002	2 (4 to 9)	0.485
Smother versus arm	**14 (12 to 17)**	**< 0.001**	**15 (12 to 18)**	**< 0.001**	**18 (15 to 21)**	**< 0.001**	**13 (10 to 17)**	**< 0.001**	**24 (20 to 28)**	**< 0.001**	**18 (13 to 23)**	**< 0.001**	**19 (14 to 24)**	**< 0.001**
Passive versus arm	**10 (8 to 13)**	**< 0.001**	**7 (5 to 10)**	**< 0.001**	**9 (6 to 12)**	**< 0.001**	**5 (2 to 8)**	**0.002**	**9 (6 to 13)**	**< 0.001**	**8 (4 to 13)**	**< 0.001**	**17 (11 to 22)**	**< 0.001**
*Legs*														
Active versus passive	**2 (0 to 5)**	**0.123**	**7 (4 to 9)**	**< 0.001**	**8 (5 to 11)**	**< 0.001**	**12 (9 to 15)**	**< 0.001**	**11 (7 to 15)**	**< 0.001**	**19 (14 to 24)**	**< 0.001**	**9 (3 to 15)**	**0.003**
Active versus smother	−2 (0 to −5)	0.079	0 (−4 to 3)	0.772	−1 (−4 to 2)	0.486	**4 (0 to 8)**	**0.045**	−2 (−6 to 1)	0.200	**10 (5 to 15)**	**< 0.001**	**7 (1 to 12)**	**0.021**
Active versus arm	**13 (11 to 16)**	**< 0.001**	**14 (11 to 16)**	**< 0.001**	**17 (14 to 20)**	**< 0.001**	**17 (14 to 19)**	**< 0.001**	**20 (17 to 23)**	**< 0.001**	**27 (23 to 31)**	**< 0.001**	**25 (21 to 30)**	**< 0.001**
Smother versus passive	**4 (2 to 7)**	**0.001**	**7 (4 to 10)**	**< 0.001**	**9 (6 to 12)**	**< 0.001**	**8 (4 to 12)**	**< 0.001**	**13 (9 to 18)**	**< 0.001**	**9 (3 to 15)**	**0.002**	2 (4 to 8)	0.484
Smother versus arm	**16 (13 to 18)**	**< 0.001**	**14 (11 to 17)**	**< 0.001**	**18 (15 to 21)**	**< 0.001**	**13 (9 to 16)**	**< 0.001**	**22 (19 to 26)**	**< 0.001**	**17 (13 to 22)**	**< 0.001**	**18 (14 to 23)**	**< 0.001**
Passive versus arm	**11 (8 to 14)**	**< 0.001**	**7 (4 to 10)**	**< 0.001**	**9 (6 to 12)**	**< 0.001**	**5 (2 to 8)**	**0.002**	**9 (5 to 12)**	**< 0.001**	**8 (4 to 12)**	**< 0.001**	**16 (11 to 21)**	**< 0.001**

*Note: p* values are also shown for each comparison, with significant differences bolded. Percentage differences more than 5% highlighted in blue.

## 4 Discussion

The purpose of this study was to compare the probability of tackler success when contacting the ball‐carrier at different legal tackle heights for different types of tackles while controlling for other tackler situational factors within seven playing levels. The key findings of this study are that from a performance perspective, contacting the shoulder versus mid‐torso at the senior levels (elite and amateur) made no difference in tackle outcomes. At the junior levels, there was a slight advantage for contacting the shoulder, which likely relates to weaker tackling technique and attempts to prevent offloads. This advantage for contacting the shoulder within the junior levels however is relatively small, and if we consider that the mid‐torso also reduces the risk of injury (Davidow et al. 2018; Tierney and Simms 2018; Tucker et al. 2017), contacting the shoulder is not recommended. Considering the drive to encourage tacklers to contact the ball‐carrier below the base of the sternum to prevent head injury risk at all levels, this study provides a timely contribution to the literature to assist coach education, law changes and tackle training programmes. For instance, governing bodies can incorporate the performance benefits as well as the safety benefits of tackling below the sternum in promoting the new tackle laws. Indeed, understanding the performance changes of any tackle injury prevention initiative, that is, focused on players' technique may enhance its perceived attributes and assist in promoting the technical recommendation, thereby potentially enhancing its overall effectiveness (Hendricks et al. 2024; Finch 2012; Hendricks, den Hollander, et al. 2015; Hendricks et al. 2012).

The probability of tackle success in relation to where the ball‐carrier is contacted varied by tackle type and within each age group. At the senior elite level, the probability of success was similar for contacting the ball‐carrier at the shoulder level versus mid‐torso for active, passive and smother tackles, whereas at the senior amateur level, tackle success probabilities were similar between the two tackle heights for active and smother tackles. Within both U18 levels, U16 and U14, contacting the ball‐carrier at the shoulder level had a higher probability of tackle success compared to the mid‐torso. This finding may relate to weaker tackling technique observed at the lower levels (Hendricks, O’connor, et al. 2015; Hendricks, den Hollander, et al. 2015; Hollander et al. 2021; Hendricks et al. 2017). Also, considering the definition of tackle success for this study, the advantage of tackling the ball‐carrier at the shoulder, compared to the other contact areas, is that it is the most effective area for preventing offloads during the tackle. In a sample of 10 matches from the 2019 Rugby World Cup, Amayo and Tierney (2021) showed that tackles at shoulder height reduced the likelihood of offloads by 91% (Amayo and Tierney 2021). The association between tackle success and contacting the ball‐carrier at shoulder height is also consistent with previous work in Super Rugby (Hendricks et al. 2014). Using a similar definition for tackle success, Hendricks et al. (2014) showed that there is a 49% higher likelihood of tackle success when contacting the ball‐carrier's shoulder area as the first point of contact (compared to contacting the mid‐torso). (Hendricks et al. 2014) Previous studies have also found that contacting the mid‐torso (centre of gravity) may reduce the risk of injury and increase the likelihood of tackle success (Davidow et al. 2018; Tierney and Simms 2018; Hendricks and Lambert 2010; Hendricks, Till, et al. 2018; Hollander et al. 2021; Hendricks et al. 2014). At the U12 level, leg tackles had a higher probability of tackle success compared to the mid‐torso irrespective of tackle type, whereas at the U14 level, leg tackles had a higher probability of tackle success for arm and passive tackles. These findings are likely an indication of the level of play, where passive and arm tackles occur more frequently (Till et al. 2023). Based on the current findings, it is evident that there are different approaches to ‘winning’ the tackle contest, which are governed by level of play. Furthermore, our study provides coaches with a better understanding of what is required to ‘win’ the tackle contest within these specific age groups. Coaches and practitioners can use this information to improve tackle training design and planning. Also, coaches can use this information to assist player development by ensuring players are equipped to meet the within age‐group tackle demands as well as the age group they are transitioning into.

Active shoulder tackles had the highest probability of tackle success across all playing levels, irrespective of contact height, whereas arm tackles had the lowest probability. From U16 onwards, the probability of tackle success did not differ between active shoulder tackles and smother tackles, suggesting that the ability to execute a safe smother tackle is as important as completing a safe active shoulder tackle, which is generally the main focus of tackle training (Hendricks, Till, et al. 2018; Hendricks et al. 2012; Hendricks et al. 2017). McIntosh et al. (2010) analysed tackle characteristics associated with injury at different levels of play (from U15 to Senior Elite) and also found that the proportion of active shoulder tackles and smother tackles increased with playing level (MCINTOSH et al. 2010). Performing active shoulder tackles and smother tackles incorrectly may lead to injury, especially head injuries and concussions (Tierney and Simms 2018; Tucker et al. 2017; Burger et al. 2016; Cross et al. 2019; Kawasaki et al. 2023). Given the probabilities of tackle success and risk of injury for these two types of tackles, dedicated efforts to train their safe techniques from a young age should be implemented. Although research and coaching guidelines exist on how to assess, monitor and design technical‐skill training for safe shoulder tackles (Hendricks, Till, et al. 2018; Hollander et al. 2021; Hendricks, Lambert, et al. 2015), guidelines for technical‐skill training of smother tackles are less prominent. Therefore, to advance tackle safety and performance, greater emphasis should be placed on the smother tackle from both a research and coaching/training perspective. For example, for the front‐on and side‐on shoulder tackle, standardised technical criteria are available to assess tackler proficiency during matches and training—which can be used by coaches and researchers. Similar standardised technical criteria are not available for the smother tackle, highlighting one potential avenue for future work in this area. In addition to how to safely perform a smother tackle, when to perform the tackle (tackle selection decision‐making) also need to be coached to reduce injury risk.

A major strength of the current study is the sample size and range of levels analysed (i.e., five age categories and two playing standards within England, New Zealand, South Africa, Portugal and USA). A standout limitation of this sample though is that it only consisted of male players. Considering the growth of women's rugby, and the general lack of research on female rugby players, there is a need to conduct a similar study across the different levels of play in the women's game (Heyward et al. 2022; Dane et al. 2022). Another caveat of the current study is that we only focused on two tackler characteristics to explain tackle success. We did not assess ball‐carrier body position (e.g., upright or bent at the waist). Also, we need to be cognisant that the tackle is a dynamic and complex skill that requires physical, technical, tactical and psychological proficiency and capacity—and present study reduced it to a handful of deterministic variables. In other words, even though tackle type and tackle height are two key characteristics associated with tackle outcomes, the interaction between physical, technical, tactical and psychological abilities also contribute to the likelihood of tackle success. Furthermore, we did not account for situational factors, which can influence player's tackling characteristics, such as match quarter, field position, match location, quality of opposition and match status (Hendricks, van Niekerk, et al. 2018). Lastly, to address the aims of the study, the tackle outcomes were dichotomised into successful and unsuccessful tackles. To further explain how technical characteristics relate to performance, future work in the area can further describe the outcomes of the tackle—for example, tackles that resulted in offloads, tackle breaks and sanctioning.

The probability of tackle success in relation to where the ball‐carrier is contacted varied by tackle type and within each age group, highlighting that there are different approaches to ‘win’ the tackle contest. Coaches and practitioners can use this information to improve tackle training design and planning within the different age groups and facilitate player development. From U16 onwards, the probability of tackle success did not differ between active shoulder tackles and smother tackles, suggesting that the ability to execute a safe smother tackle is as important as completing an active shoulder tackle, which is generally the main focus of tackle training. To advance tackle safety and performance, greater emphasis should be placed on how to perform the smother tackle under the new laws.

## Conflicts of Interest

Potential conflicts of interest for the authors include Sean Scantlebury and Cameron Owen's Research Fellowships are part‐funded by the Rugby Football League. Nicholas Gill is employed by New Zealand Rugby Union. Simon Kemp and Keith Stokes are employed by the Rugby Football Union. Ross Tucker is employed by World Rugby. Ben Jones is employed by Rugby Football League and Premiership Rugby in a consultancy capacity. Sharief Hendricks acts as a research consultant for World Rugby. Sharief is an Associate Editor for EJSS and social media editor.
